# An Ang-1-releasing self-assembling peptide coating for inflammation modulation and suppression of smooth muscle proliferation

**DOI:** 10.3389/fbioe.2026.1815590

**Published:** 2026-06-22

**Authors:** Shuyao Wang, Tao Ye, Kai Lan, Chunbin Wang, Jin Wang, Xin Li, Zhen Zhang

**Affiliations:** 1 Department of Cardiology, The Third People’s Hospital of Chengdu, College of Medicine, Southwest Jiaotong University, Chengdu, Sichuan, China; 2 Department of Cardiology, The Affiliated Hospital of Southwest Jiaotong University, The Third People’s Hospital of Chengdu, Cardiovascular Disease Research Institute of Chengdu, Chengdu, Sichuan, China

**Keywords:** angiopoietin-1, anti-inflammation, cardiovascular implant materials, endothelialization, RADA16 polypeptide

## Abstract

Post-stent implantation vascular complications, primarily characterized by impaired re-endothelialization and persistent inflammatory responses, frequently result in delayed vascular healing and subsequent adverse clinical outcomes, including late-stage thrombosis and in-stent restenosis. Cytokines act as master regulators of vascular repair, coordinating endothelial regeneration, inflammatory responses, and tissue remodeling. Angiopoietin-1 (Ang-1) is a particularly significant regulatory molecule within the vascular system, exerting pleiotropic effects on vascular homeostasis through dual regulation of endothelial regeneration and vascular stabilization. Self-assembling peptide systems, with their unique combination of programmable molecular design and inherent biocompatibility, are revolutionizing targeted vascular therapy development. In this study, we developed an RADA16 peptide-based coating loaded with Ang-1 to mimic the unbound state of endogenous Ang-1, thereby modulating inflammatory responses, promoting re-endothelialization, and accelerating vascular repair. The RADA16 peptide coating enabled sustained Ang-1 release for more than 14 days. Both the peptide coating and Ang-1-loaded peptide coatings exhibited excellent cytocompatibility. The Ang-1 loaded coating significantly enhanced the growth and migration of human umbilical vein endothelial cells (HUVECs) while selectively inhibiting the proliferation of smooth muscle cells (SMCs). Furthermore, the coating effectively suppressed macrophage (MA) proliferation and reduced secretion of pro-inflammatory cytokines, including interleukin-6 (IL-6) and tumor necrosis factor-α (TNF-α). Meanwhile, both PAs coating and PAs-Ang coating promote the polarization of macrophages toward the M2 phenotype, facilitating inflammation resolution and tissue repair. The Ang-1 loaded coating exerting anti-inflammatory effects and creating an immune-favorable microenvironment conducive to vascular repair. The Ang-1-eluting peptide coating exhibited a trifecta of therapeutic effects by selectively promoting endothelial cell proliferation and migration while suppressing inflammatory responses. This multifunctional bioactive coating reveals anti-inflammatory activity, pro-endothelialization capacity, and inhibition of smooth muscle cell hyperplasia. This work offers a novel strategy for cardiovascular biomaterials development, and provides a new surface modification approach for cardiovascular implants.

## 1 Introduction

The increasing of cardiovascular diseases has led to growing demand for cardiovascular medical devices. However, current cardiovascular interventional devices face several challenges, including thrombosis, chronic inflammation, and restenosis (Mukheja et al., 2024). As a major class of cardiovascular devices, vascular stents are particularly limited by issues such as delayed reendothelialization, in-stent restenosis, and chronic inflammation (Wu et al., 2024). Surface modification of biomaterials has emerged as an important approach for vascular stent enhancement, as it could introduce various bioactive substances onto material surfaces without altering the substrate’s structure or mechanical properties (Preetam et al., 2025; Zhu et al., 2025). This technique can effectively endow materials with multiple biological functionalities, offering a promising solution for current clinical challenges of vascular stents.

It is crucial for addressing delayed re-endothelialization for vascular stents (Cherian et al., 2022; Kang et al., 2025). Surface functionalization represents a primary strategy for promoting endothelialization on cardiovascular device surfaces (Wang et al., 2025a). This technology could endow material surfaces with unique selectivity and affinity for endothelial cells (Li et al., 2024), as well as promoting the homing of endothelial progenitor cells (Lu et al., 2025). Various approaches, such as immobilizing or loading antibodies, biological factors (Xiao et al., 2025), peptides (Cortese et al., 2025), or nucleic acids onto stent surfaces, can selectively enhance the adhesion and growth of endothelial cell on materials surface, thereby accelerating the endothelialization process (Anitha et al., 2026).

Angiopoietin-1 (Ang-1), a key member of the angiopoietin family (Yan et al., 2022). Ang-1 together with VEGF serves as an endothelial cell-specific growth factor in vascular systerm (Zhao et al., 2025). Ang-1 acts synergistically with VEGF to promote vascular endothelial cell proliferation, survival, and inhibition of apoptosis, while supporting vascular system formation and development (Sanchez et al., 2025; Zhang et al., 2025). Currently, Ang-1 is an important therapeutic target for panvascular diseases (Wallace et al., 2021). In the body, Ang-1 exists in two forms: a fraction remains freely diffusible within the vascular tissue (Tsigkos et al., 2003), while other binds to the extracellular matrix (McClennan and Hoffman, 2024). These two forms coexist in normal vasculature. Studies indicate that Ang-1’s diverse biological functions depend not only on its intrinsic properties but also on its existing form (Saharinen et al., 2017). Our previous work successfully immobilized Ang-1 onto vascular stent surfaces to simulate its bound state (Li et al., 2015). This Ang-1 immobilized surface preserved the bioactivity of Ang-1 and significantly promoted the endothelialization of vascular tissue.

Self-assembling peptides are a class of short peptide molecules with the capability of spontaneously forming ordered nanostructures through intermolecular interactions (Rosa et al., 2023). Self-assembling peptides possess unique properties, including natural origin, simple and rapid assembly, structural stability, and programmable amino acid sequences (Hiew et al., 2023). Wen et al. (2014) discovered that the AEAEAKAKAEAEAKAK peptide can spontaneously assemble into nanofibers and nanofiber hydrogels. It could mimic the three-dimensional structure of extracellular matrix to promote cell adhesion and proliferation. The RADA16 peptide (Yu et al., 2024) also demonstrates self-assembly capability and pH/ion-mediated gelation properties (Sankar et al., 2021), with excellent biocompatibility making it suitable for applications such as 3D tissue culture materials (Laigle et al., 2026). Boopathy and Davis, (2014). co-loaded platelet-derived growth factor (PDGF) and fibroblast growth factor-1 (FGF-1) into RADA16 peptide hydrogels. These two growth factors could be co-releaseed from the RADA16 peptide hydrogels. *In vivo* results showed the growth factor loaded hydrogel could significantly reduce the infarct size and increase vascular density, at 4–8 weeks (Wang et al., 2021). Using a similar approach, Li et al, (2017) employed RADA16 for VEGF loading and controlled release. The VEGF loaded RADA16 hydrogel could significantly increase the density and diameter of neovascularization after 28 days. Guo et al. engineered the RADA peptide by incorporating an LRKKLGKA sequence (heparin-binding domain) at its terminus. This modified peptide effectively controlled VEGF release for over 28 days. Significant improvements in angiogenesis, cardiac function, and tissue remodeling were observed *in vivo*, for the VEGF loaded RADA16 hydrogel group (Guo et al., 2012).

**SCHEME 1:**
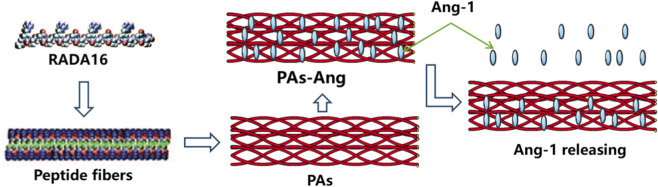
Schematic diagram of PAs peptide coating preparation and Ang-1 loading and elution.

In this study, we construct one RADA16 peptide coating onto vascular stent material surfaces and loaded with Ang-1 to create an Ang-1 peptide eluting surface, as shown in Scheme 1. This system mimics the unbound state of Ang-1 in the vascular microenvironment, and releases Ang-1 to target vascular to promote vascular repair. This Ang-1 loaded coating effectively promotes endothelial cell growth and migration while selectively inhibiting vascular smooth muscle cell proliferation and macrophage-mediated inflammatory responses. Ang-1 loaded peptide coating shows promising potential to accelerate endothelialization of vascular implants, suppress neointimal hyperplasia, and regulate vascular inflammation. This work provides a novel solution for cardiovascular device development to reduce complications after implantation of the stents.

## 2 Materials and methods

### 2.1 Materials

316L stainless steel (SS) was obtained from Dabo Steel Co., Ltd. Angiopoietin-1 (Ang-1), RADA16 peptide, Absolute ethanol (Chengdu Kelong Chemical Co., Ltd.), Cell Counting Kit-8 (CCK-8) (Dojindo Molecular Technologies), IL-6/TNF-α ELISA kits (Sigma-Aldrich), 0.25% Trypsin, Fetal bovine serum (FBS), Cell culture medium (Gibco).

### 2.2 RADA16 peptide pre-assemble

10 mg of RADA16 peptide was dissolved in 1 mL deionized water (DI water) and sonicated for 30 min. After sterilization, the solution was pre-assembled at 4 °C for 1 week and stored.

### 2.3 RADA16 peptide coating preparation

The pre-assembled RADA16 peptide solution was sonicated for 15 min, and then mixed with an equal volume of DI water, and sonicated again for 5 min to obtain 5 mg/mL RADA16 solution. Then, 50 μL RADA16 solution was uniformly coated onto a 10 mm-diameter stainless steel (SS) substrate and dried in air for 6 h. This sample was labeled as PAs.

### 2.4 Ang-1 loaded RADA16 peptide coating preparation

The pre-assembled RADA16 solution was sonicated for 15 min, mixed with equal volume of 600 ng/mL Ang-1 aqueous solution, and then vortexed for 5 min to achieve 300 ng/mL Ang-1 RADA16 solution. Then, 50 μL Ang-1 RADA16 solution was uniformly coated onto a 10 mm-diameter SS substrate and dried in air for 6 h. This sample was labeled as PAs-Ang.

### 2.5 Characterization of RADA16 coatings

The surface morphology of the RADA16 coating was examined using scanning electron microscope (SEM) (Quanta 200 electron scanning microscope of Philips) and digital camera. The thickness of the coating was detected by using the scratch method. A scratch was made on the coating surface, and the height difference between the coating and the scratch bottom was measured via laser confocal microscopy (CLSM, A1R+).

The water absorption rate of the coatings is detected by the gravimetric method. Uncoated substrate weight (*m*_*0*_) was recorded, and then coated substrate was also recorded (*m*_*1*_). After immersing in water for 24h, the coated substrate was also detected (*m*_*2*_). Water absorption rate was calculated as shown in Equation 1:

$$W\text{ater }A\text{bsorption}\left(\%\right)=\frac{m2-m1}{m1-m0}\times 100\%$$ (1)

The RADA16 coated samples were dried at 60 °C for 24 h and weighed (*m*_*3*_). Then, the samples were immersion in DI water at 37 °C for 1, 3, 7, or 14 days. After that, samples were dried at 60 °C for 24 h and reweighed (*m*_*4*_). Mass loss rate was calculated as shown in Equation 2:

$$M\text{ass }L\text{oss}\left(\%\right)=\frac{m3-m4}{m3-m0}\times 100\%$$ (2)

The surface wettability of the coatings was assessed at room temperature using a Krüss GmbH DSA 100 MK2 drop shape analyzer.

### 2.6 *In vitro* release profile of Ang-1

The release behavior of Ang-1 from the coatings were quantified using a human Ang-1 ELISA kit. The sterilized PAs-Ang samples were immersed in 1 mL sterile PBS at 37 °C for 6 h, 12 h, and 1, 3, 7, and 14 days. At each time point, 10 μL of the incubation medium was collected for analysis according to the protocol of the Ang-1 ELISA kit.

### 2.7 Hemocompatibility evaluation of RADA16 coatings

#### 2.7.1 Platelet activation test

Fresh human blood was centrifuged at 1,500 rpm for 15 min to obtain platelet-rich plasma (PRP). The fresh human blood used in this study was donated by volunteers. The study was approved by the Ethics Committee of Southwest Jiaotong University, and all volunteers signed informed consent forms. 400 μL PRP was added to the surface of each sample and then incubated at 37 °C for 45 min. After that, non-adherent platelets were removed by PBS washing, and then the washed samples were incubated with 2.5% glutaraldehyde overnight. Subsequently, these samples were dehydrated with 50%, 75%, 90%, and 100% ethanol solution. Then, after drying treatment, the platelet morphology was observed by SEM.

#### 2.7.2 Platelet adhesion test

After incubation with PRP for 1h, the samples were washed, and stained with 5 μg/mL rhodamine 123 for 15 min at 37 °C. And the adhered platelet was observed via fluorescence microscopy.

#### 2.7.3 APTT assay

Platelet-poor plasma (PPP, 300 μL) was incubated with samples at 37 °C for 30 min. The activated partial thromboplastin time (APTT) of the PPP after treated with different samples was measured using a coagulation analyzer (ACL200, Instrumentation Laboratory Co., United States).

### 2.8 *In vitro* cytocompatibility of RADA16 coatings

Before cell-based assays, all PAs coatings were sterilized. Briefly, the RADA16 peptide stock solution was filter-sterilized to remove bacteria, and the subsequently prepared RADA peptide coatings were further sterilized by ultraviolet (UV) irradiation.

HUVECs (human umbilical vein endothelial cells [purchased from Merck Life Science - EndoGRO®]) were maintained at 37 °C ± 0.5 °C in a humidified atmosphere of 5% ± 0.5% CO_2_ and 95% ± 2% relative humidity, and were seeded onto the surface of each sample at density of 3 × 10^4^ cells/mL, and onto sterilized samples. Additionally, prior to cell seeding, HUVECs were serum-starved for 24 h in DMEM containing 1% serum, under conditions of 37 °C ± 0.5 °C and 5% ± 0.5% CO_2_. After 1- and 3-day culture, cell viability was assessed using CCK-8 kit at 450 nm. Then, the cells cultured on the surface were stained with rhodamine 123 and observed using fluorescence microscope. Subsequently, culture medium of each sample was collected and stored at −20 °C for ELISA quantification of tissue factor (TF) and PGI_2_.

A transwell system was used to measure the migration of HUVECs. For details, the samples were placed under the transwell chamber, and HUVEC suspension (1 × 10^5^ cells/mL) was added into the chamber. After 16 h culture, the migrated cells from the chamber to sample surface were stained with crystal violet and observed by microscope.

Smooth muscle cells (SMCs, purchased from ScienCell Research Laboratories) were maintained at 37 °C ± 0.5 °C in a humidified atmosphere of 5% ± 0.5% CO_2_ and 95% ± 2% relative humidity. (1 × 10^4^ cells/mL, M199 medium) and Macrophages (MAs, RAW264.7, purchased from InvivoGen) were maintained at 37 °C ± 0.5 °C in a humidified atmosphere of 5% ± 0.5% CO_2_ and 95% ± 2% relative humidity, and were used at passages 8–10. (5 × 10^4^ cells/mL, DMEM): were cultured as per HUVEC protocol. Prior to co-culture with the materials, SMCs and MAs were also subjected to serum starvation for 24 h following the same method as described for endothelial cells. After cultured for 24 h, MAs culture medium was analyzed via ELISA kits (mouse IL-6, TNF-α, IL-10, and VEGF kits, Sigma). After cultured for 48 h, MAs were collected and then analyzed by flow cytometry (Flow cytometer model: LongCyte C2080, Beijing LayerWise Biotechnology Co., Ltd.) to identify M1 and M2 phenotypes. (M1:CD68, iNOS; M2:CD68, CD206) (CD68 (mouse mAb, BioLegend, #137007), iNOS (rabbit pAb, Proteintech, #18985-1-AP), secondary antibody: PE-goat anti-rabbit IgG (MCE, #HY-P89168)).

### 2.9 Statistical analysis

All quantitative assays in this study were performed with at least three independent samples. The data were analyzed in a one-way analysis of variance (ANOVA), date are presented as mean ± standard deviation. The statistically significant was regarded as follows: P < 0.05, *; P < 0.01, ** and P < 0.001, ***.

## 3 Results

### 3.1 Material characterization of RADA16 coatings

**FIGURE 1:**
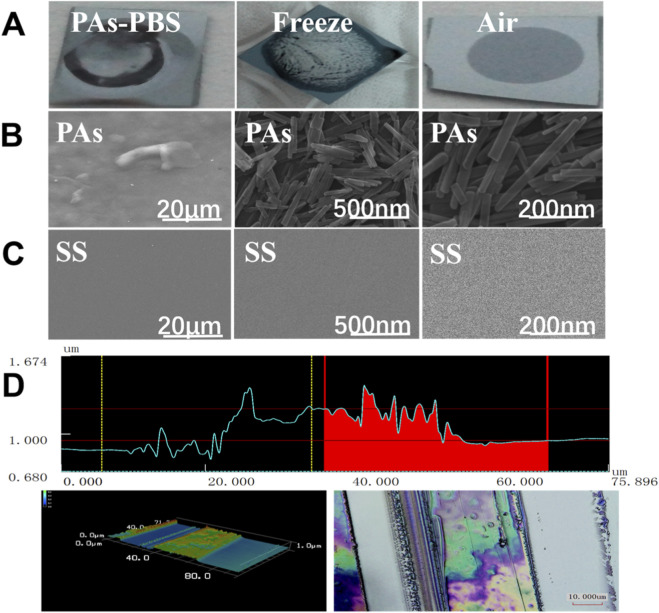
**(A)** Digital photos of peptide coatings formed under different conditions. From left to right: RADA16 peptide hydrogel onto the silicon surface; RADA16 coating formed by freeze-drying; RADA16 coating formed by air-drying; **(B)** SEM images of peptide coatings prepared by air-drying method; **(C)** SEM image of stainless steel coating; **(D)** Thickness determination of polypeptide coatings via CLSM, 3D reconstruction image of the coating, and image of fibers on the coating surface.

The RADA16 peptide coatings formed on silicon wafer surfaces under different conditions were shown in Figure 1 After pre-assembly, the RADA16 solution formed a distinct hydrogel upon PBS treatment, which exhibited a loose and porous structure after freeze-drying. In contrast, the pre-assembled RADA16 solution, after direct air-drying, did not form a loose and porous structure; instead, it coated the material surface evenly. Subsequently, the surface morphology of the air-dried RADA16 peptide coating was observed by SEM. The air-dried RADA16 coating consists predominantly of peptide-based nanowires and nanosheets, with diameters averaging ∼100 nm and lengths spanning 300–1000 nm. The PAs coating was composed of stacked nanosheet-like structures. For the SS substrate, the surface was relatively flat without such nanosheet-like structures (Figures 1A-C).

Figure 1D shows micrographs of the coating surface. From the surface micrograph and three-dimensional reconstruction, it can be observed that the coating surface is uneven with significant height variations. Meanwhile, based on the three-dimensional and two-dimensional reconstruction results, the coating thickness was determined to be approximately 1 μm, as shown in Table 1. Subsequently, the water absorption rate of the coating was measured. As presented in Table 1, the water absorption rate of the coating was approximately 8.8%. Therefore, the coating does not rehydrate to revert to a hydrogel state and can be used for constructing coatings on biomaterial surfaces. That is, during coating formation, the peptides undergo further assembly into sheet-like or wire-like nanostructures, leading to a substantial reduction in pore volume and consequently lower water absorption capacity.

**TABLE 1:** Water absorption rate and thickness of RADA16 peptide coating.

Sample	Water absorption rate (%)	Thickness (μm)
PAs	8.8 ± 2.99	1.036 ± 0.039

**FIGURE 2:**
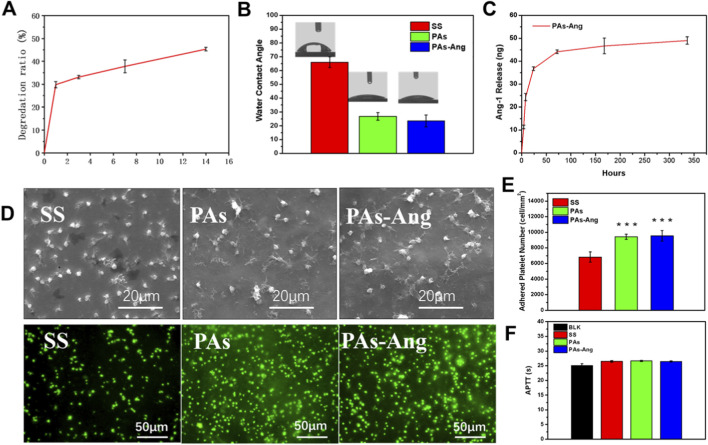
**(A)** *In vitro* degradation ratio of RADA16 polypeptide coating; **(B)** Water contact angles of SS, PAs, and PAs-Ang surfaces; **(C)** *In vitro* release profile of Ang-1; **(D)** SEM and fluorescence images of platelet adhesion on the surface of SS, PAs, and PAs-Ang groups; **(E)** statistical results of platelet adhesion number; **(F)** results of APTT for each group. (Statistically significant was regarded as follows: P < 0.05, *; P < 0.01, ** and P < 0.001, ***, N = 5).

The stability of the peptide coating was evaluated, as shown in Figure 2A. The mass loss rate of the coating in aqueous solution, was about 30% within the first day. Subsequently, the mass loss rate stabilized at nearly 7% per-week, and the total mass loss of the coating was less than 50% over 14 days. This may be attributed to the spontaneous assembly process of the peptide. In the coating, the larger nanostructures such as nanowires/nanosheets, smaller nanofibers may both coexist. These smaller fibers are likely to dissolve into the aqueous solution during the start-up stage, leading to the large mass loss on the first day. Subsequently, the mass loss rate tended to stabilize. Based on results of the capability and stability of the peptide coatings discussed above, the RADA16 peptides can form coatings on the surface of materials through simple air drying, and these coatings can degrade in aqueous solutions.

The hydrophilicity/hydrophobicity of the peptide coating was characterized by water contact angle (Figure 2B). Compared to the SS substrate, water contact angles of the peptide coating and Ang-1-loaded peptide coating were significantly reduced. Notably, the water contact angles of the peptide coating before and after Ang-1 loading showed no difference, suggesting that Ang-1 did not alter the surface wettability of the peptide coating. The increase in hydrophilicity of the peptide coating may result from two factors: surface chemistry properties and surface morphology. The peptide coating contains abundant polar functional groups (e.g., -NH_2_ and -COOH), which enhance the surface hydrophilicity compared to the SS surface. SEM results revealed that surface roughness of the peptide coating increased compared to the flat SS substrate. The improved hydrophilicity of the peptide coating may be attributed to the complementary effects of polar group interactions and morphological alterations.

### 3.2 *In vitro* release of Ang-1 from peptide coatings

Figure 2C shows the *in vitro* release profile of Ang-1. A significant burst release of Ang-1 was observed within the first 3 days, with over 30% of Ang-1 released within the first 24 h. After day 3, the release transitioned to a stable, slow-release phase, and the cumulative release rate remained below 50% by the end of the 14-day period.

### 3.3 *In vitro* anticoagulation evaluation of ang-1-loaded coatings

Cardiovascular implantable devices must interact with both human tissues and the circulatory system upon implantation, necessitating excellent tissue compatibility and blood compatibility for blood-contacting implants. The blood compatibility of PAs before and after Ang-1 loading was evaluated. Figure 2D presents SEM images of the adhered platelets after 45min incubation, and the rhodamine fluorescence staining results of platelet adhesion after 1h. The SS surface exhibited extensive platelet adhesion, with platelets displaying spreading and pseudopodia extension. Similarly, both PAs coatings and Ang-1-loaded PAs coatings showed substantial platelet adhesion, including fully spread platelets and fibrous aggregates on the surface, likely formed by fibrin clotting due to fibrinogen activation.

Rhodamine fluorescence staining and statistical analysis (Figure 2E) revealed that the number of adherent platelets on both PAs and Ang-1-loaded PAs surfaces was significantly higher than on SS. However, no notable difference in platelet adhesion was observed between the PAs and Ang-1-loaded PAs coatings. Activated partial thromboplastin time (APTT) results (Figure 2F) demonstrated that SS and both peptide coatings had minimal impact on APTT compared to blank plasma, suggesting that the PAs coatings and their degradation products do not significantly interfere with the intrinsic coagulation pathway.

In summary, compared to SS, the peptide coating promotes platelet adhesion and activation, yet exhibits minimal impact on the intrinsic coagulation pathway. Consequently, the anticoagulant properties of the peptide coating are inferior to those of the SS substrate, and it’s *in vivo* application may pose a thrombotic risk. However, the peptide coating can be functionalized by incorporating bioactive molecules or drugs. To address its insufficient blood compatibility, anticoagulant agents (e.g., heparin, nitric oxide donors) could be loaded into the coating to enhance its hemocompatibility for cardiovascular applications.

### 3.4 *In vitro* cytocompatibility assessment of ang-1-loaded coatings

**FIGURE 3:**
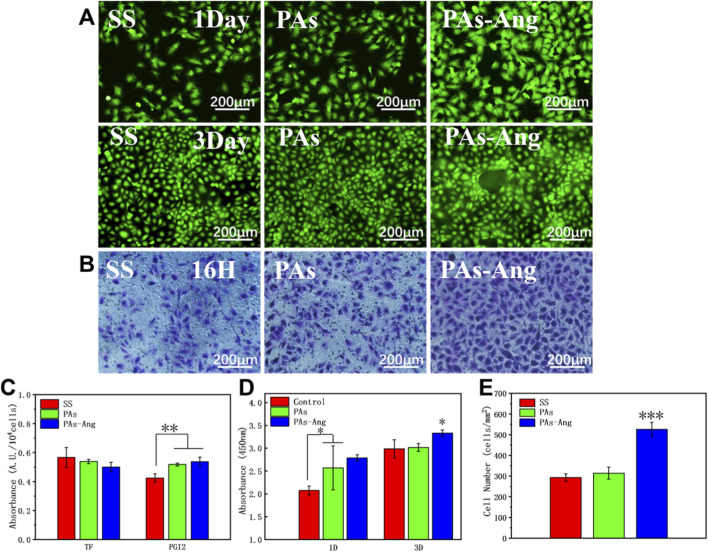
**(A)** fluorescence images of HUVECs grown on the surface of each group; **(B)** crystal violet staining image of endothelial cell migration; **(C)** The release amount of TF and PGI_2_by HUVECs grown on the surface of each group; **(D)** CCK-8 assay results of HUVECs grown on the surface of each group; **(E)** statistical results of migration quantity. (Statistically significant was regarded as follows: P < 0.05, *; P < 0.01, ** and P < 0.001, ***, N = 5).

Endothelial cells, as the natural vascular barrier, play a critical role in inhibiting coagulation and maintaining blood flow patency (Puy et al., 2024). Endothelial dysfunction or injury caused by atherosclerosis or interventional therapies disrupts vascular stability, leading to plaque progression and adverse events post-stent implantation (Wang et al., 2025b). Thus, endothelial cell growth behavior directly determines vascular homeostasis and the efficacy of interventional treatments. Figures 3A,D show the results of endothelial cell proliferation (CCK-8 assay) and rhodamine fluorescence staining, respectively. After 1 day of culture, both the peptide coating and Ang-1-loaded peptide coating surfaces exhibited significantly higher endothelial cell viability and density compared to the SS control. By day 3, all surfaces were nearly confluent with endothelial cells. However, the CCK-8 values for the Ang-1-loaded coating remained significantly higher than those for SS and the unloaded PAs. The comparable cell density and viability between SS and PAs at day 3 may be attributed to contact inhibition among cells on confluent surfaces. These results demonstrate that the peptide coating promotes endothelial cell growth and proliferation, with Ang-1 loading further enhancing this effect. This improvement likely stems from the sustained release of Ang-1 from the coating into the culture medium, stimulating endothelial proliferation.

Endothelial cells on material surfaces may exhibit functional dysregulation due to material-induced stimuli or microenvironmental changes, altering their secretion profiles (Costa et al., 2025). Figure 3C quantifies the secretion of TF and PGI_2_ by endothelial cells cultured for 1 day. While no significant differences in TF secretion per cell were observed across the three groups, both peptide coatings (with and without Ang-1) significantly increased PGI_2_ secretion per endothelial cell compared to SS. These findings suggest that: None of the materials induced pro-thrombotic TF overexpression. The peptide coatings, particularly when loaded with Ang-1, enhanced PGI_2_ secretion, which may promote vasodilation and improve blood flow *in vivo*, thereby offering beneficial effects on vascular homeostasis.

The Ang-1-loaded peptide coating gradually releases Ang-1 into the aqueous environment, establishing a concentration gradient that may induce directional migration of surrounding cells toward the coating surface. To investigate the effect of Ang-1-loaded coatings on the three-dimensional migratory behavior of vascular endothelial cells, a transwell migration assay was conducted. Figure 3B displays crystal violet-stained images of endothelial cells that migrated to the lower chamber, while Figure 3E quantifies the number of migrated cells. The PAs-Ang group exhibited nearly complete coverage of the lower chamber surface by migrated endothelial cells, with a significantly higher cell count compared to the SS and unloaded PAs groups. This indicates that Ang-1 released from the PAs-Ang coating into the culture medium effectively drives chemotactic migration of endothelial cells toward the coating. These results demonstrate that Ang-1 released from the peptide coating creates a chemoattractant gradient, promoting directional migration of endothelial cells in three-dimensional space. Such behavior could enhance the recruitment of endothelial cells to the coating surface post-implantation, thereby accelerating endothelialization of cardiovascular stents and improving their clinical performance.

**FIGURE 4:**
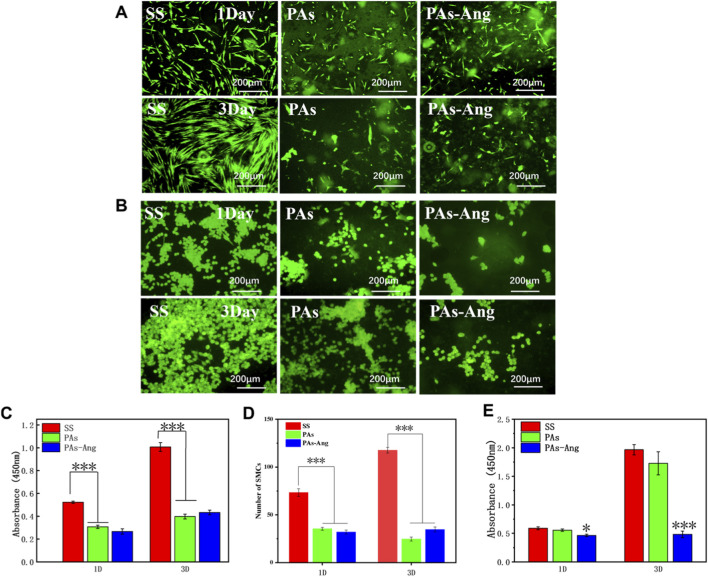
Proliferation of SMCs and MAs on SS, PAs, and PAs-Ang samples: **(A)** fluorescence images of SMCs grown on the surface of each group; **(B)** CCK-8 assay results of SMCs grown on the surface of each group; **(C)** statistical results of SMCs number grown on the surface of each group; **(D)** fluorescence images of MAs grown on the surface of each group; **(E)** CCK-8 assay results of MAs grown on the surface of each group. (Statistically significant was regarded as follows: P < 0.05, *; P < 0.01, ** and P < 0.001, ***, N = 5).

Cardiovascular stents have significantly improved the quality of life for patients with atherosclerosis (Zhang and Wei, 2021). However, long-term clinical outcomes reveal persistent challenges, notably late-stage thrombosis and restenosis (Saidi-Seresht et al., 2025). Restenosis primarily arises from excessive proliferation of SMCs, leading to neointimal hyperplasia (Jain et al., 2021). Consequently, the impact of implant materials on SMC growth behavior is closely tied to the clinical efficacy of vascular stents。The effects of peptide coatings (with and without Ang-1 loading) on SMCs growth were evaluated, as shown in Figures 4A,C,D. Rhodamine fluorescence staining and CCK-8 assay results indicate that after 1 day of culture, SMCs density on both PAs and PAs-Ang coatings was markedly lower than on the SS substrate. SMCs on SS exhibited an elongated spindle-shaped morphology, whereas those on PAs coatings appeared significantly shortened, and SMCs on PAs-Ang coatings displayed a mixed morphology (predominantly elongated spindles with some oval or shortened spindle shapes). By day 3, SMCs on the SS surface showed substantial proliferation and increased cell density. In contrast, SMCs on PAs and PAs-Ang coatings exhibited minimal proliferation, adopting an aggregated and contracted morphology. Meanwhile, the quantitative analysis of SMCs also revealed that the number of SMCs grown on the surfaces of PAs and PAs-Ang was significantly lower than that on SS. Furthermore, after 3 days of culture, the difference in SMC numbers between the PAs coating and SS surfaces further widened, while SMCs on both PAs and PAs-Ang surfaces showed almost no proliferation. Prior studies suggest that such aggregated cells may undergo apoptosis in later growth stages. This inhibitory effect on SMC proliferation may stem from the nanowire/nanosheet architecture of the peptide coating, which likely hinders SMC adhesion and spreading. These findings demonstrate that the peptide coating effectively suppresses SMC proliferation, potentially mitigating neointimal hyperplasia and reducing the risk of stent-related restenosis.

The inflammatory response of the peptide coatings was evaluated *in vitro* by analyzing macrophage growth behavior and cytokine secretion on the coated surfaces. Figures 4B,E present rhodamine-stained fluorescence images and CCK-8 quantitative results of macrophages cultured for 1 day and 3 days, respectively. Macrophages on the PAs coating formed aggregated clusters, while the number of adherent macrophages on the Ang-1-loaded PAs coating (PAs-Ang) was significantly lower than on both PAs and SS. At both time points, macrophages on all surfaces exhibited a rounded morphology with no observable activation-related morphological changes (e.g., elongation or pseudopodia formation). CCK-8 results revealed that macrophages on SS and PAs coatings underwent active proliferation, with marked increases in cell number and viability over time. In contrast, macrophages on the PAs-Ang coating showed minimal proliferation, maintaining low cell counts throughout the 3-day culture period. This suppression of macrophage growth on the Ang-1-loaded coating may be attributed to the sustained release of Ang-1 into the culture medium, which likely exerts inhibitory effects on macrophage proliferation. These findings suggest that the Ang-1-loaded peptide coating effectively suppresses macrophage proliferation, potentially mitigating macrophage-mediated inflammatory responses *in vivo*. This property could enhance the biocompatibility of cardiovascular implants by reducing adverse inflammatory reactions post-implantation.

**FIGURE 5:**
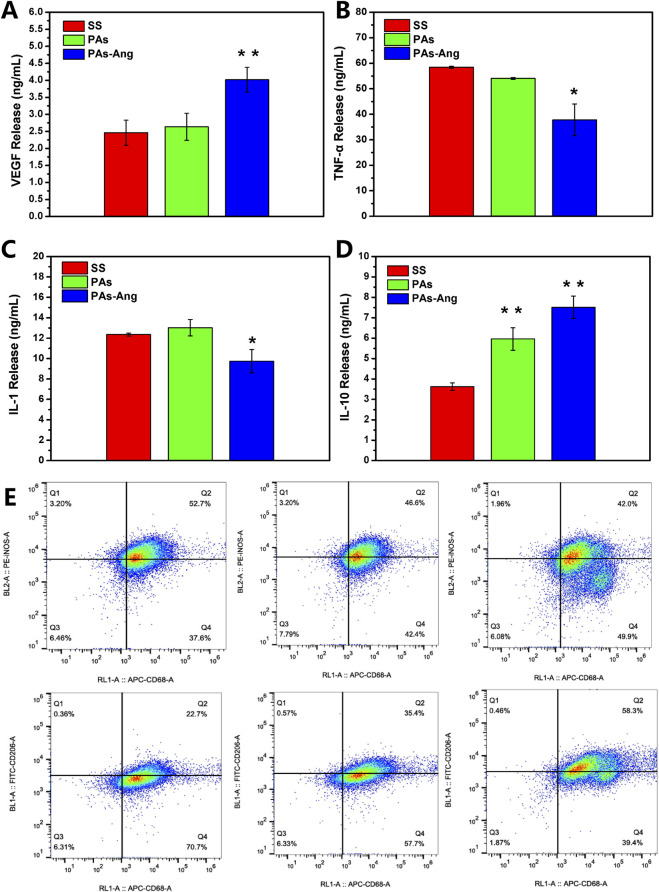
Expression of growth factors and inflammatory factors by MAs growing on SS, PAs, and PAs-Ang samples **(A)** VEGF; **(B)** TNF-α; **(C)** IL-1; **(D)** IL-10; **(E)** Flow cytometry analysis of macrophages cultured on SS, PAs, and PAs-Ang samples. (Statistically significant was regarded as follows: P < 0.05, *; P < 0.01, ** and P < 0.001, ***, N = 5).

As shown in Figures 5A-D, Macrophages secrete diverse cytokines in response to microenvironmental changes, including pro-inflammatory factors (e.g., IL-1β, TNF-α) and pro-repair factors (e.g., VEGF, IL-10) (Mao et al., 2026). The balance between these cytokines critically determines whether inflammation escalates or resolves into tissue healing (Xue et al., 2024). The secretion levels of these cytokines by macrophages cultured on the material surfaces for 1 day were quantified, as shown in Figure 5. For pro-inflammatory cytokines: IL-1β and TNF-α secretion showed no significant difference between the SS and unloaded PAs coating. However, macrophages on the Ang-1-loaded PAs-Ang exhibited significantly lower levels of IL-1β and TNF-α compared to both SS and PAs. For pro-repair cytokines:VEGF secretion by macrophages on the PAs-Ang coating was markedly higher than on SS and unloaded PAs. Both PAs and PAs-Ang coatings induced significantly elevated IL-10 secretion compared to SS. These results suggest that the Ang-1-loaded peptide coating not only suppresses pro-inflammatory cytokine production but also enhances the secretion of pro-repair mediators, potentially steering the inflammatory response toward tissue repair rather than chronic inflammation. Meanwhile, flow cytometry was employed to investigate the effect of PAs coatings on macrophage polarization. As shown in Figure 5E, the PAs coating increased the expression of CD206 on the macrophage surface, decreased the expression of iNOS, and promoted macrophage polarization toward the M2 phenotype. Moreover, due to the sustained release of Ang-1 from PAs-Ang, CD206 expression was further increased, iNOS expression was further reduced, and macrophage polarization toward the M2 phenotype was further enhanced. These results indicate that both PAs and PAs-Ang coatings can effectively regulate macrophage polarization while inhibiting the secretion of inflammatory cytokines, demonstrating their potential to modulate tissue inflammation and promote tissue repair.

## 4 Discussion

In this study, we developed an Ang-1-loaded self-assembling RADA16 peptide coating and systematically evaluated its biological functions. The results demonstrated that this coating not only supports sustained Ang-1 release but also integrates the promotion of endothelial cell growth and migration, inhibition of smooth muscle cell proliferation, and immunomodulation through macrophage M2 polarization. These properties may address some limitations of current cardiovascular implants, such as delayed re-endothelialization, excessive neointimal hyperplasia, and chronic inflammation. Ang-1 released from the peptide coating binds to the endothelial cell-specific receptor Tie2, thereby activating downstream signaling cascades including PI3K, FAK, and ERK. These pathways coordinately regulate Rac1-and Cdc42-mediated cytoskeletal rearrangement and focal adhesion dynamics, ultimately driving the directional chemotactic migration of endothelial cells (Kim et al., 2000). In addition, free Ang-1 can directly serve as an adhesion substrate for integrins, synergistically enhancing cell migration via a Tie2-independent pathway (Carlson et al., 2001). Meanwhile, both the Ang-1 encapsulated within the peptide coating and that released from it can bind to Tie2, triggering receptor dimerization and autophosphorylation, which in turn activate downstream core signaling pathways such as PI3K/Akt and ERK1/2. These signals coordinately regulate cell survival and cell cycle progression, thereby effectively promoting vascular endothelial proliferation (Kanda et al., 2005). Our previous study also demonstrated that Ang-1 immobilized on material surfaces promotes both proliferation and migration of vascular endothelial cells (Li et al., 2015). Furthermore, Ang-1 is an important regulator of vascular inflammation. Ang-1 loaded in or released from the peptide coating exerts anti-inflammatory effects via the NF-κB pathway upon binding to Tie2 (Gu et al., 2010). Moreover, Ang-1 modulates macrophage polarization toward the M2 phenotype through the PHD2-TIE2 positive feedback loop (Hamm et al., 2013), which is of great significance for vascular tissue repair.

In addition, the PAs coating exhibits an Ang-1-independent function in inhibiting SMC proliferation. This is likely attributable to the fact that the PAs coating consists of numerous peptide nanosheets. The abundant nanosheets on the coating surface restrict cell spreading and reduce cytoskeletal tension, promoting the cytoplasmic retention of key mechanoresponsive molecules YAP/TAZ and reducing their nuclear translocation as well as the transcription of proliferative genes such as Cyclin D1 (Lee et al., 2025). Simultaneously, the nanosheet topography induces remodeling of focal adhesion complexes, leading to the formation of smaller and more dispersed focal adhesions, which reprograms FAK signaling output and diminishes pro-proliferative signaling intensity (David et al., 2025). Furthermore, the nanosheet surface topography differentially activates the RhoA/ROCK pathway, enhances cytoskeletal contractility, and upregulates the expression of contractile phenotype markers such as α-SMA and calponin, thereby promoting the switch of SMCs from a synthetic/proliferative phenotype to a contractile/quiescent phenotype (Chaterji et al., 2014). These multi-level mechanisms act synergistically to achieve effective inhibition of smooth muscle cell proliferation (Lin et al., 2022).

In summary, the Ang-1-loaded RADA16 self-assembling peptide coating integrates inflammation regulation, pro-endothelialization, and inhibition of smooth muscle cell proliferation (anti-proliferative) functions, representing a promising strategy for constructing surface coatings for cardiovascular implants. This work not only confirms the multiple regulatory roles of the Ang-1/Tie-2 signaling pathway combined with the peptide coating in endothelial cell migration, macrophage polarization, and suppression of smooth muscle cell proliferation, but also reveals the potential mechanism by which peptide-based biomaterials synergistically regulate cell behavior through the integration of biochemical and biophysical signals.

## 5 Conclusion

In conclusion, an Ang-1-loaded self-assembling RADA16 peptide coating has been successfully developed. The coating enables sustained release of Ang-1 and exhibits multiple bioactive functions: it promotes endothelial cell growth and migration, PGI_2_ expression, inhibits smooth muscle cell proliferation, and suppresses macrophage proliferation while driving their polarization toward the anti-inflammatory M2 phenotype, thereby reducing pro-inflammatory cytokine secretion (IL-1, TNF-α) and effectively modulating the inflammatory response. Collectively, this coating integrates inflammation regulation, pro-endothelialization, and anti-proliferative capabilities, offering a promising surface modification strategy for cardiovascular implants. By accelerating endothelial regeneration, modulating macrophage-mediated inflammation, and inhibiting smooth muscle hyperplasia, this coating provides a novel approach for the development of advanced surfaces for cardiovascular implantable devices.

## Data Availability

The original contributions presented in the study are included in the article/supplementary material, further inquiries can be directed to the corresponding authors.
